# The Potential of Plants to Absorb Xenobiotics

**DOI:** 10.3390/plants13070922

**Published:** 2024-03-22

**Authors:** Fiore Capozzi, Valeria Spagnuolo

**Affiliations:** Department of Biology, Campus Monte S. Angelo, University of Naples Federico II, Via Cinthia 4, 80126 Napoli, Italy

## 1. Introduction

Environmental pollution is a pressing problem that endangers our biosphere. It particularly affects densely populated and industrialized areas, where society is experiencing drastic changes in terms of land use. Among the myriad pollutants that undermine the health of our ecosystems every day, non-biodegradable substances are causing the greatest concern [1]. These pollutants accumulate in the environment because of natural and anthropogenic activities, poisoning water bodies, sediments, soils, and the atmosphere [2,3]. Xenobiotics, such as heavy metals, PAHs, and POPs, can accumulate along with microplastics in the organisms that determine biomagnification along the food chain, seriously compromising the health of all living beings, including humans [4,5]. Understanding how plants interact with and react to these pollutants, and therefore monitoring and cleaning the environment affected by these substances, is crucial to minimizing risks to ecosystems. To date, many different approaches have been used to solve environmental contamination, with different degrees of effectiveness. The use of plants to monitor and restore polluted environments could be an effective and eco-friendly method for both biomonitoring and phytorestoration. Below, we present the results of recent scientific research, contributing to the expansion of our knowledge on the ability of plants to absorb/adsorb xenobiotics from the environment.

## 2. An Overview of Published Articles

The articles in the present collection can be classified according to three main topics: (1) the use of plants for biomonitoring purpose; (2) utilizing plants for the phytorestoration of polluted sites; and (3) the effects of pollutants on plant health.

### 2.1. Plant and Biomonitoring (3 Contributions)

The potential of using moss as a bioindicator was indagated by Zinicovscaia et al. (2021) and Sorrentino et al. (2021). Zinicovscaia et al. (2021) (contribution nr.1) who evaluated the deposition of potentially toxic elements in the Republic of Moldova. In particular, they analysed multi-elemental accumulation in samples of the native moss *Hypnum cupressiforme* collected from 41 sampling sites. The authors compared data collected in 2020 with a survey from 2015 to 16 and found a significant decrease in the elemental content of Cr, As, Se, Br, Sb, Cd, Pb, and Cu. Moreover, a comparison with the elemental content of the moss collected in the neighbouring countries highlighted Moldova’s higher content of As, Al, Ni, V, Cr, and Fe, and lower content of Cd and Pb. The authors suggested that vehicular traffic, industry, power plants, and mining were the main sources of pollution.

The potential of using mobile moss-bags (i.e., bags filled with the moss *H. cupressiforme* fixed to bicycles) to intercept airborne particles and related elements was evaluated by Sorrentino et al. (2021, contribution nr.5). In this study, seven volunteers transported moss-bags in triplicate for 50 days on their bike while commuting to work and returning home in the city of Antwerp (BE). In addition to moss sensors, one bicycle was fitted with a PM sampler and was driven along four routes characterized by different land uses: urban, industrial, green route, and the total path. The authors found that the gradient of pollution was consistent with land use (urban, industrial, and green areas). In particular, the authors found higher elemental accumulation in the industrial route, with the lowest levels detected in the green route. Interestingly, the elemental accumulation detected in the mobile system was higher than that found in a previous experiment carried out in the same study area using fixed moss-bags.

In the research (contribution nr. 4) of Abdullahi Bala Alhassan et al. (2021), the potential use of mangrove *Avicennia marina* was evaluated for the biomonitoring of rare earth elements in the central Red Sea. The authors evidenced higher concentrations of rare earth elements at Al Lith and South Jeddah mangrove ecosystems. Among the rare earth elements detected, lutetium had the highest bioconcentration factor in *Avicennia marina*. There was evidence of the strong contamination of sediments with La, Ce, Pr, Sm, Gd, Ho, Er, and Yb, and moderate contamination with Tm, Tb, and Dy was detected as well.

### 2.2. Plant and Phytoremediation (5 Contributions)

Four research articles were contributed by Verasoundarapandian et al. (2021), Puasa et al. (2022), El Berkaoui et al. (2022), Chamba-Eras et al. (2022), and Landi et al. (2022) on the use of plants in the purification of different environmental matrices. These are presented here, respectively, as contributions 6, 7, 8, 10, and 12.

As known, the marine environment can be profoundly damaged by oil spills. To counteract these events, Verasoundarapandian et al. (2021) evaluated a filter-based system made with coconut waste. The selectivity of coco peat sorbent was tested using coco peat, coco fibre, and a peat–fibre mix. The results evidenced that peat was the best diesel sorbent, minimizing seawater uptake. The authors also found that heating treatment improved the diesel sorption capacity of coco peat. In order to solve the same challenge (i.e., diesel spill pollution), Puasa et al. (2022) tested fibres from empty fruit bunches of oil palms at different experimental conditions in terms of temperature, time, packing density, and diesel concentration. The authors optimized the sorption condition using a one-factor-at-time (OFAT) approach, and the results indicated that all tested conditions significantly affected diesel sorption. The predicted model was validated using the statistical response surface methodology (RSM) and finally successfully applied in a laboratory experiment, producing results that were totally consistent with the predictions.

Data have provided by El Berkaoui et al. (2022) on the use of plants for the remediation and phytostabilization of mining residuals. The authors selected species among the spontaneous vegetation found around an abandoned pyrrhotite mine in Marrakech (Morocco) in order to test their applicability for site recovery. Among the nine species collected, five species showed robust metal bioconcentration and translocation capacity, demonstrating a high tolerance to trace metals. Consequently, these species can be considered good candidates for the phytoremediation of the study area.

Gold mines in southern Ecuador are sources of heavy metals. Chamba-Eras et al. (2022) evaluated two native woody plants to assess their ability to restore mine soil from cadmium, lead, zinc, and mercury. The authors collected *Erato polymnioides*, *Miconia* sp., and rhizosphere soil from a mining site and in a proximal natural area as a control. After soil and plant tissue analyses, they found that *Miconia* sp. was a good candidate for the phytostabilization of Cd and Zn; *E. polymnioides* performed better for the phytoextraction of Cd and Zn; and both species were effective in Hg phytoextraction. The authors concluded that the two species are well adapted to the edapho-climatic conditions of the region and that their potential for phytoremediation can be boosted by increasing their biomass.

The bioremediation potential of the green algae *Caulerpa racemosa* was evaluated by Landi et al. (2022) in a laboratory trial in which the algae were grown in the presence of zinc. This metal had detrimental effects on the species, decreasing its growth and photochemical efficiency. However, *C. racemosa* demonstrated efficient Zn uptake capacity.

### 2.3. Effects of Nutrients and Xenobiotic on Plants (4 Contributions)

The effects of various nutrients and xenobiotics on plants are the objects of the articles by Tkachenko et al. (2021), Leitão et al. (2021), Sorrentino et al. (2022), and Kalinhoff et al. (2022), i.e., contributions 2, 3, 9 and 11, respectively.

Manganese is a micronutrient necessary for plants. However, exposure in high amounts can negatively affect the development of plants, with this species exerting an especially toxic effect on acid soil. Tkachenko et al. (2021) screened the major Mn-tolerant crops in the collection of barley (*Hordeum* L., Poaceae) held by the Russian Institute of Plant Genetic Resources. They found that plants with the highest numbers of manganese-resistant genotypes belonged to the Russian Federation, Sweden, Finland, and the northwestern regions of the CIS countries. These barley cultivars can be considered good sources of acid-resistant barley germplasms.

The research of Leitão et al. (2021) evaluated the uptake of acetaminophen (ACT)—a pharmaceutical found in soil and irrigation water—by lettuce plants growth in hydroponics. The authors studied antioxidant activity and found that leaves contaminated with ACT showed a decrease in glutathione reductase, along with an increase in anthocyanin directly proportional to the exposure time and ACT concentration.

In the paper by Sorrentino et al. (2022), the authors studied the effects of Pb and Cd on cardoon plants grown in hydroponic conditions. The authors estimated the genotoxicity induced by metals using ISSR molecular markers, and evaluated the distribution of the metal fractions between the symplast and apoplast in 3 cultivars of *Cynara cardunculus* var. altilis (L.) (i.e., Sardo, Siciliano, and Spagnolo). In line with the literature reports, lead generated a higher degree of genotoxic damage compared to Cd in all cultivars. However, a cultivar-specific response occurred, with Spagnolo showing a higher degree of genome template stability compared to the other cultivars. The author observed that, under conditions of metal stress, this cultivar developed smaller and more numerous epidermal cells, providing a larger wall surface area and thereby allowing a wider metal sequestration in the apoplast, thus reducing the intracellular metal fraction.

The effect of mercury (Hg) on the germination and development of *Bidens pilosa*, *Taraxacum officinale* (Asteraceae), and *Heliocarpus americanus* (Malvaceae) was studied by Kalinhoff et al. (2022). These species were selected from among ecotypes adapted to gold mining sites in south of Ecuador. The results provided by these plants were compared with those of *Lactuca sativa* (Asteraceae), which is known for its Hg tolerance. Hg treatments mainly affected the germination in *T. officinale*, with the next largest effect seen in *B. pilosa*. The exposure to higher concentrations of Hg caused an increase in T50 in *B. pilosa* and a reduction in *H. americanus*. All examined species showed a reduction in radicle length when exposed to the highest concentration of Hg, which was especially severe in *L. sativa*. Therefore, under Hg contamination, the grass *B. pilosa* and the tree *H. americanus* could possesses greater establishment and survival potential than lettuce.

## 3. Conclusions

The articles presented in this collection revealed the variety of research areas in which plants can be used in order to monitor and counteract pollution in the environment. This opportunity is based on the ability of plants to absorb/adsorb pollutants. However, plant–pollutant interactions may also affect plant growth, altering morphological traits and physiological pathways. Therefore, it is especially important to investigate plant responses to environmental pollution. These responses are indeed species-specific and dose/pollutant-dependent; hence, their study is of paramount importance for setting up effective plant-based protocols for use in the biomonitoring and remediation of polluted environments. Finally, this collection provides new valuable data to support the use of plants for these purposes, while at the same time laying the foundation for future research.
